# An Unexpected Etiology of Pancreatic Panniculitis: A Case Report

**DOI:** 10.1089/pancan.2016.0021

**Published:** 2017-01-01

**Authors:** Anna C. Evans, Aatur D. Singhi, Herbert J. Zeh, Nathan Bahary, Randall E. Brand

**Affiliations:** ^1^ Division of Gastroenterology, Hepatology, and Nutrition, University of Pittsburgh School of Medicine, UPMC Presbyterian, Pittsburgh, Pennsylvania.; ^2^ Department of Pathology, University of Pittsburgh School of Medicine, Pittsburgh, Pennsylvania.; ^3^ Division of Surgical Oncology, Department of Surgery, University of Pittsburgh Medical Center, Pittsburgh, Pennsylvania.; ^4^ Department of Medical Oncology, University of Pittsburgh Medical Center, Pittsburgh, Pennsylvania.

**Keywords:** acinar cell cancer, pancreatic panniculitis, pancreatic-portal fistula

## Abstract

**Background:** Pancreatic panniculitis is a rare cause of subcutaneous fat necrosis secondary to elevated serum levels of pancreatic enzymes. It is most often associated with pancreatic acinar cell carcinoma, but has also been seen in patients with pancreatitis.

**Case report:** We present a case of a 64-year-old Caucasian man without symptoms of pancreatitis, who presents with pancreatic panniculitis manifesting in multiple subcutaneous ulcerating nodules of the bilateral lower extremities, discovered to have a previously unreported etiology for this condition. He had no evidence of pancreatitis or malignancy, but instead a pancreatic-portal fistula resulting in panniculitis.

**Conclusion:** Peripancreatic vascular lesions must also be considered in the differential diagnosis of pancreatic panniculitis. The diagnosis, pathology, and treatment of pancreatic panniculitis are reviewed herein.

## Introduction and Background

Pancreatic panniculitis is a rare cause of subcutaneous fat necrosis in patients with pancreatic acinar cell carcinoma: it has also rarely been associated with other pancreatic malignancies and chronic pancreatitis.^1^ Here, we describe a case of pancreatic panniculitis associated with a rare cause of hyperlipasemia.

## Presentation of Case

A 64-year-old male with a history of hypertension, hyperlipidemia, and coronary artery disease presented to the hospital with 3 weeks of worsening lower extremity subcutaneous nodules. He first noted the nodules several days after a fall, in which he struck the extensor surfaces of his extremities, but had no skin tears, bruising, or trauma to the head or torso. Over the following 3 weeks, he noted both an increasing number and worsening ulceration in the nodules.

He denied abdominal pain, nausea, vomiting, diarrhea, or constipation. He did have a “pins and needles” sensation in bilateral lower extremities, and 10 lbs of weight loss over the preceding 3 weeks. He saw his primary care medical doctor who referred him to dermatology for evaluation of the skin lesions. The dermatologist, suspecting panniculitis, ordered blood work, which revealed a lipase level >6000 U/L (Reference Range 0–160 U/L). The patient had a history of past alcohol use with ∼750 mL of vodka per day for 5–6 years. He quit ∼5 years before this presentation and was abstinent for 3 years: ∼2 years before presentation he resumed drinking 1–2 mixed drinks per day.

**FIG. 1.**
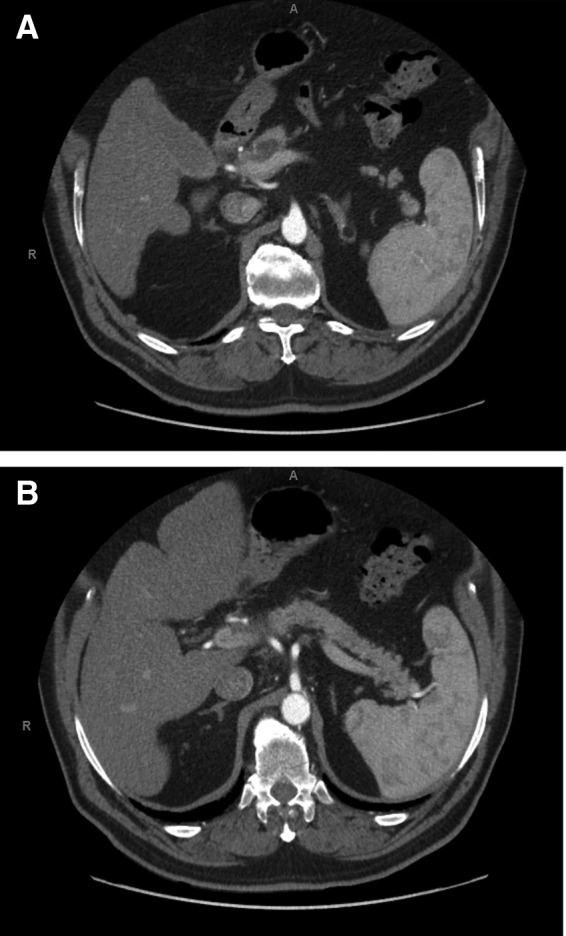
**(A, B)** 1.8 cm water attenuation rounded lesion in neck of pancreas anterior to splenic-portal confluence. **(A)** Along right lateral aspect of this lesion in adjacent cuts is a subtle hypoattenuation involving the pancreatic parenchyma, which extends near the hepatic artery (which is almost completely encased) **(B)** and a partially occlusive portal vein thrombus.

On physical examination at presentation to this hospital, the patient's abdomen was soft and nontender. Multiple erythematous ulcerated nodules were seen on his bilateral lower extremities extending to his thighs. A computed tomography of the abdomen revealed a 1.8 cm hypoattenuating lesion in the neck of the pancreas anterior to the portal confluence and encasing the adjacent portion of the hepatic artery (Fig. 1A, B). Enlarged 1 cm lymph nodes were seen adjacent to the hepatic artery. A nonocclusive thrombus in the proximal main portal vein adjacent to the hypoattenuating lesion was concerning for possible tumor thrombus. A magnetic resonance cholangiopancreatography showed a possible cystic lesion in the pancreatic genu with upstream pancreatic ductal dilatation. Suspicion for a pancreatic acinar cell carcinoma was high with these imaging findings and associated skin findings of pancreatic panniculitis.

**FIG. 2.**
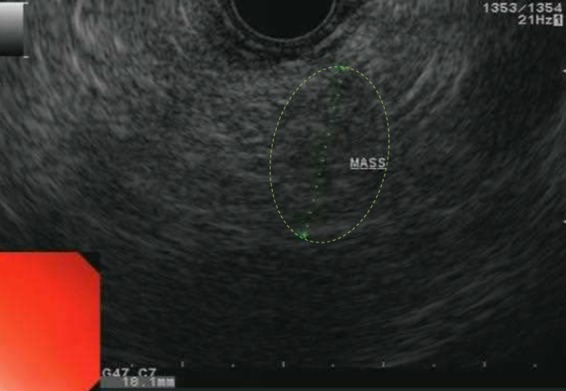
Endoscopic ultrasound. Isoechoic, heterogeneous, mixed solid, and cystic mass in the neck of the pancreas. Largest dimensions 23 mm by 21 mm.

**FIG. 3.**
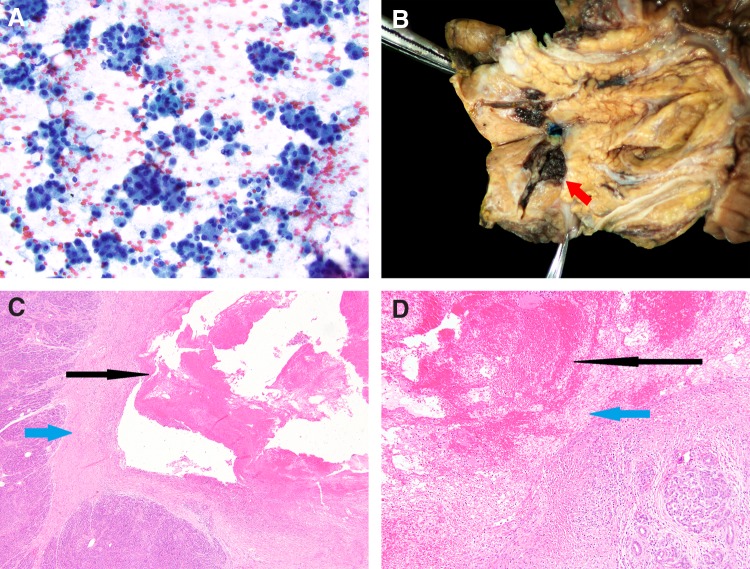
**(A)** Fine needle aspiration with cytopathology revealed reactive appearing acinar cells and no evidence of a malignant neoplasm. **(B)** Resection of the pancreatic lesion revealed the presence of a pancreatic-portal fistula with associated thrombus (red arrow). **(C, D)** This finding was confirmed histologically with communication of the portal vein and adjacent pancreatic parenchyma. The fistula was characterized by hemorrhage (black arrows) and surrounding fibrosis (blue arrows) with residual pancreatic parenchyma at the periphery.

An endoscopic ultrasound (EUS) revealed an isoechoic, heterogeneous, mixed solid, and cystic mass in the neck of the pancreas, which measured 23 mm by 21 mm (Fig. 2). Fine needle aspiration (FNA) with subsequent cytopathology revealed benign-appearing, reactive acinar cells (Fig. 3A). Repeat EUS with FNA revealed several ductal-like cells, macrophages, and lymphocytes. Lymph node biopsy showed scattered lymphocytes. Lipase and amylase measurements done at time of this procedure were 6205 U/L (Reference Range 22–51 U/L) and 3052 U/L (Reference Range <90 U/L Normal), respectively.

Although FNA was nondiagnostic for malignancy, concern for a pancreatic acinar cell carcinoma was still high. An oxaliplatin-based chemotherapy regimen for acinar cell carcinoma was considered, but could not be offered given the lack of confirmatory cytology. A robotic-assisted classic pancreaticoduodenectomy was performed with resection of the pancreatic head and *en bloc* resection of the portal vein, superior mesenteric vein, and splenic vein confluence. The surgical specimen did not demonstrate a malignant process, but rather a pancreatic-portal vein fistula (Fig. 3B) with an associated thrombus. These findings were confirmed histologically and showed a clear communication between the pancreatic parenchyma and portal vein with associated hemorrhage and fibrosis (Fig. 3C, D). No findings of a pancreatic neoplasm or acute pancreatitis were seen within the remaining pancreatic parenchyma. The patient's rash receded quickly after surgical resection. He gradually returned to a low-fat diet accompanied by one to two capsules of pancreatic enzyme supplementation before each meal. At 6 months after surgery, the patient was feeling well and had returned to his work and normal daily activities.

## Discussion

To our knowledge, this report offers the first case of a pancreatic-portal fistula occurrence without a clinical diagnosis of chronic pancreatitis, as well as the first report of panniculitis secondary to a pancreatic-portal fistula. Without evidence of pancreatitis, but with imaging evidence for a pancreatic mass, the most appropriate therapy was removal of the suspected mass. Discovery of the vascular nature of the mass after pathologic examination and its associated abnormal blood flow explained the elevated pancreatic enzymes and associated panniculitis, as well as the characteristic imaging findings with lack of confirmatory malignant cytology. It is unclear whether the patient may have had prior episodes of asymptomatic pancreatitis resulting in pseudocyst formation and subsequent fistulization: there are unfortunately no prior data points in the patient's medical history to provide support for this. Rare reports of pancreatic-portal fistulas in the past have reported these anomalies in the context of chronic pancreatitis^2–4^: our patient had no signs of this diagnosis either before or after his operation. Specifically, on the resected pancreatic parenchyma there was no evidence of chronic pancreatitis.

Histologically, pancreatic panniculitis is characterized by subcutaneous fat necrosis, acute and chronic inflammation, and hemorrhage due to systemic release of pancreatic enzymes.^5^ Our patient had elevations in pancreatic amylase and lipase due to communication between the portal vein and pancreatic duct in the pancreatic-portal fistula. Following adipocyte necrosis and saponification, there is infiltration of neutrophils, which produces a light-colored thick fluid often seen oozing from characteristic skin lesions. Histology will usually reveal “ghost cells” under the microscope-necrotic, anucleated adipocytes.^6,7^ Our patient had undergone biopsy by the dermatologist he saw outside our hospital: the biopsies were not made available but reportedly showed fat necrosis and panniculitis.

Clinical association of pancreatic disease associated with panniculitis has been mainly made with pancreatic acinar call carcinoma,^8–10^ but others have been reported in association with pancreatitis^11^ and extrapancreatic neoplasms.^6^ Although it is an extremely rare condition affecting only 0.3–1.0% of patients with pancreatic disease, its pathogenesis remains mysterious. Over 130 unique cases of pancreatic panniculitis have been reported in the literature over the past 20 years.^6^ Importantly, many patients manifest skin nodules before the related pancreatic disease has been recognized,^5^ making this an important diagnostic herald or even potential trigger for evaluation of pancreatic abnormalities. Treatment of pancreatic panniculitis primarily consists of treating the underlying pancreatic disease. In many cases, this consists of chemotherapeutic or surgical treatment of a pancreatic malignancy, or supportive treatment of the patient's pancreatitis.

## Conclusion

In conclusion, elevations in pancreatic enzymes causing dermatopathologies may have multiple etiologies. Malignancies, inflammation, and vascular lesions must be considered as underlying problems in an effort to treat the patient's primary pancreatic disease for relief of cutaneous manifestations.

## Abbreviation Used

EUS: endoscopic ultrasound
FNA: fine needle aspiration
